# Dataset on the Marine Sustainability in the United Arab Emirates

**DOI:** 10.1016/j.dib.2020.105742

**Published:** 2020-05-22

**Authors:** Osman Gulseven

**Affiliations:** Skyline University College, Sharjah, UAE

**Keywords:** Sustainability, Life Below Water, Marine Life, Coastal Ecosystems

## Abstract

This data compiles the relevant indicators on measuring the UAE's attainments towards sustaining marine life and coastal ecosystems. Those indicators are compiled from three databases, namely from the United Nations, Bertelsmann-Stiftung (BS) Foundation, and the Ocean Health Index (OHI). While the UN and BS indicators are widely accepted in measuring sustainability, many of the indicators in these databases are ambiguous and incomplete. The data from OHI is complete and offers a better perspective on measuring the quality of life below water at a country level. This is an interesting case study, which can extend to other countries. The compiled data can be used to make better decisions for future sustainability initiatives in protecting marine life. Interpretation of this data can be found in the article by Gulseven (2020) titled "Measuring achievements towards SDG 14, *life below water*, in the United Arab Emirates" [1].

Specifications table

**Table:** 

Subject	Agricultural and Biological Sciences
Specific subject area	Ecology, Evolution, Behavior, and Systematics
Type of data	Table
	Figure
How data were acquired	Data is compiled from three publicly available resources that offer indicator-based assessments. These resources are the UN United Nations, Bertelsmann-Stiftung (BS) Foundation, and the Ocean Health Index (OHI).
Data format	Raw
	Analyzed
Parameters for data collection	The UN declared 17 Sustainable Development Goals to be measured by more than 200 indicators. Indicators relevant to marine life are listed under SDG 14, life below water. Those indicators are filtered according to Nationality.
Description of data collection	Data includes several indicators collected from three primary sources, namely the United Nations (UN), Bertelsmann-Stiftung (BS) Foundation, and the Ocean Health Index (OHI). Data is redesigned for cross-comparison. Only data on the United Arab Emirates are provided here.
Data source location	Country: United Arab Emirates
	Latitude and longitude: Between 22°30′ to 26°10′ N and 51° to 56°25′ E
Data accessibility	Data is uploaded to Mendeley Data (https://data.mendeley.com/datasets/pf9sk7hbjd/1)
Related research article	Gulseven, O. (2020) 'Measuring achievements towards SDG 14, life below water, in the United Arab Emirates', *Marine Policy*. Elsevier. doi: 10.1016/j.marpol.2020.103972.[1]

## Value of the data

The dataset allows for comparative analysis of marine sustainability indicators as defined by the United Nations, Bertelsmann-Stiftung indicators, and Ocean Health Index.
The dataset can be used by policymakers and environmental decision-makers to assess the current state of the marine ecosystems.
National and supranational environmental policies can be redesigned to improve the quality of marine ecosystems using this dataset.
The data can serve as a basis for further research on forecasting trends in coastal sustainability indicators.

## 1 Data Description

**Fig. 1:**
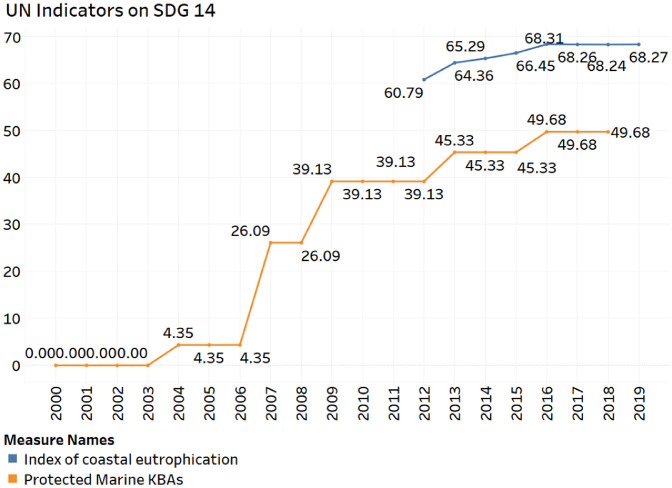
The ratio of Protected Marine Key Biodiversity Areas and Index on Coastal Eutrophication

**Fig. 2:**
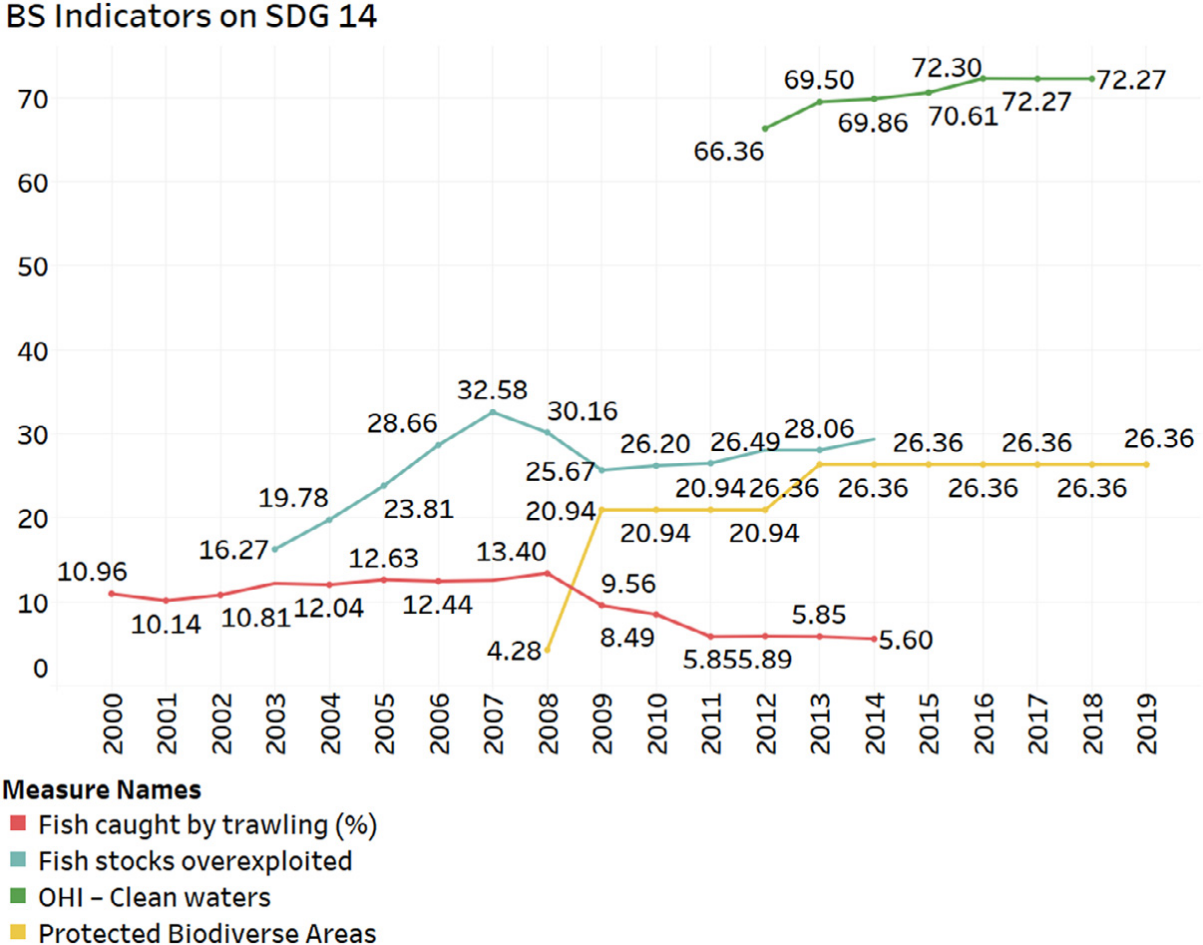
BS Indicators on SDG 14

**Fig. 3:**
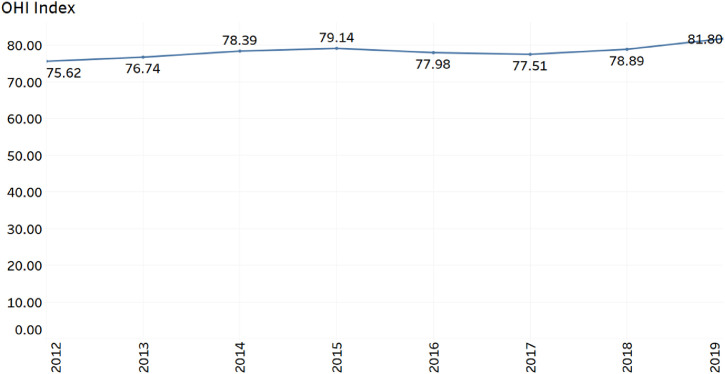
OHI index on Ocean Health

**Fig. 4:**
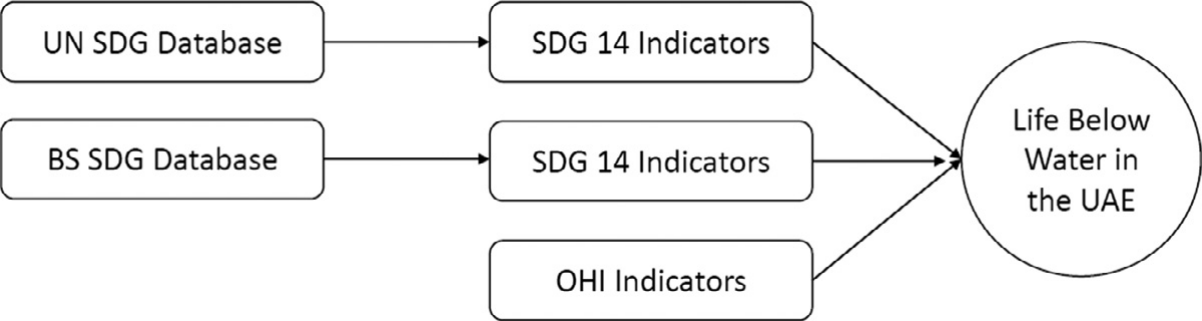
Data Filtering Process

**Fig. 5:**
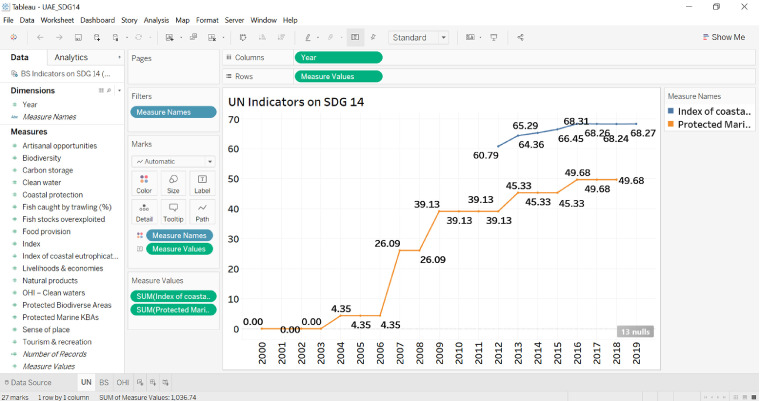
Tableau interface snapshot

This data shows the sustainability performance of the United Arab Emirates (UAE) in achieving sustainable development goal 14 (SDG 14), *life below water*. Fig. 1 describes the recent trends in the protected marine key biodiversity areas and coastal eutrophication index. Fig. 2 shows the marine sustainability indicators. Table 1 shows the evolution of indicators used to create the Ocean Health Index (OHI). Fig. 3 visualizes the recent trends in the OHI index. Fig. 4 shows the conceptual data filtering process. Fig. 5 shows the actual Tableau software interface snapshot that filters data. Only the UAE sustainable development indicators relevant to SDG 14 are presented in the data.

**Table 1:** UAE Indicators used in the Calculation of Ocean Health Index

Year	Artisanal opportunities	Biodiversity	Coastal protection	Carbon storage	Clean water	Food provision	Livelihoods & economies	Natural products	Sense of place	Tourism & recreation
2012	100	93.93	93.16	91.52	60.79	57.96	100	46.25	69.99	42.61
2013	100	93.9	93.13	91.58	64.36	60.49	100	46.12	66.88	50.92
2014	100	93.86	93.22	91.68	65.29	61.74	100	57.24	68.44	52.46
2015	100	93.82	93.19	91.7	66.45	62.25	100	57	69.38	57.63
2016	100	93.8	93.13	91.75	68.31	62.31	100	41.01	71.21	58.25
2017	100	93.68	93.15	91.72	68.26	59.18	100	42.42	71.09	55.64
2018	100	93.59	93.06	91.66	68.24	59.58	100	53.99	70.66	58.07
2019	100	93.52	93.13	91.71	68.27	59.59	100	81.67	70.22	59.88

In 2015, the United Nations announced 17 SDGs to be achieved by 2030. These goals have a broad scope, ranging from eliminating poverty and improving healthcare, to sustaining biodiversity while retaining economic growth. The partnership is also an essential aspect of SDGs [2].

SDG 14 aims to protect marine life by providing a blueprint for managing coastal and ocean ecosystems. The theme of this SDG is the sustainable use of marine ecosystem services, which started giving alarm signals in recent years [3]. A significant amount of the population depends purely on marine resources as their primary source of livelihood [4]. The decline in the sustainable fish population needs to be reversed by feasible means such as regulations [5] and quota allocation to achieve optimal viable yield [6]. There is a need for circular economic systems to avoid land-based pollution from ending up in the oceans [7]. Marine life is threatened by pollution [8] – specifically plastic pollution [9]. Those emerging issues motivated the UN to declare eight targets and ten indicators to measure the achievements towards SDG 14 [10]. All signatory nations are expected to contribute to achieving this goal in collaboration with national and supranational entities.

Similar to other Gulf states, the UAE is a marine state where life has started and is continuing along the coastal strip. Almost 90% of the population of 10 million is living in the narrow coastal strip that extends from Abu Dhabi to Ras Al Khaimah. As such, protecting the sustainability of marine life in this area is of uttermost importance [11]. This is a part of the sustainable wildlife initiative in the country that aims to protect wildlife biodiversity while providing economic growth. However, it is a significant challenge to measure the achievements towards these goals objectively. One approach is to use the UN database on sustainability indicators. The UN defined indicators are as follows:

- Index of coastal eutrophication and floating plastic debris density
- Sustainable Management of Marine and Coastal Ecosystems (missing)
- Average Marine Acidity (Ph) (missing)
- Sustainable Fish Stock (missing)
- The average proportion of Marine Key Biodiversity Areas (KBAs) covered by protected areas (%)
- Degree of international instruments (missing)
- Sustainable fisheries (missing)
- Scientific marine research (missing)
- Equal access to fishing (missing)
- Signatures on Law on Sea (binary)

Out of those ten indicators, only 3 are available for the UAE. UAE is a signatory to the Law on Sea. The other two indicators are shown in Fig. 1.

Fig. 1 shows that there has been a significant increase in the proportion of protected marine key biodiversity areas. Twenty years ago, there was no area under legal protection. As of 2019, almost 50% of the key marine biodiversity areas are protected by environmental law. Coastal eutrophication has also improved slightly during the last decade. The coastal eutrophication score has increased from 60.79 to 68.27 between 2012 and 2019. This suggests an improvement of 12% during this period.

### 1.1 Bertelsmann-Stiftung Data

Another source of data to measure achievements towards SDG 14 is developed by the Germany-based foundation Bertelsmann-Stiftung (BS). This index offers explicitly a ranking system for measuring SDG attainments based on a relatively compact set of indicators [12,13]. The list of SDG 14 indicators in the BS database are as follows:

- Mean area that is protected in marine sites important to biodiversity (%)
- Ocean Health Index Goal – Clean waters
- Percentage of fish stocks overexploited or collapsed by EEZ
- Fish caught by trawling (%)

The easiness of comparison between nations increased the popularity of this database. In recent years, it has almost become a semi-official benchmark to assess national SDG achievements. Nevertheless, as can be seen in Fig. 2 below, the data also has its drawbacks and is not available for all periods

Trawling has a horrible impact on the seafloor. It does not just catch all kinds of fish, but can destroy the entire seafloor ecosystem. Fig. 2 shows that the ratio of fish caught by trawling has declined by half from 10.96% in 2000 to 5.60% in 2014. The proportion of protected marine areas important for biodiversity has also increased to 26.36%. The clean water index had also improved from 66.36 in 2012 to 72.27 in 2018. On the negative side, there is an increase in the overexploitation of fish. Thus, the fish stock in the country's marine ecosystems is depleting due to overcatching.

### 1.2 Ocean Health Index (OHI) Data

The Ocean Health Index (OHI) is a collaborative teamwork with inputs from several NGOs. The most visible outcome of this collaboration is a centralized database that reports indicators on the state of the oceans from more than one hundred sources. In order to provide a comparative analysis for countries, ten indicators are combined to create an index. This index is made public on the website of the project [14]. The most recent data suggests that the UAE has an OHI index score of 82 and is ranked 19^th^ among 221 territories listed. This data is in existence long before the UN announcement on SDGs. The current list of variables used in calculating the OHI index is as follows (all available for UAE):

- Artisanal opportunities
- Biodiversity
- Coastal protection
- Carbon storage
- Clean water
- Food provision
- Livelihoods & economies
- Natural products
- Sense of place
- Tourism & recreation

Table 1 below offers a recent analysis of the data on the OHI indicators as below:

According to Table 1, the UAE has consistently received the top score (100) in terms of artisanal opportunities and livelihoods & economies indicators. Biodiversity, coastal protection, carbon storage, and sense of place scores were also stable around 93.5, 93.1, 91.7, 70.2. There are also significant improvements in clean water (60.8 to 68), food provision (58 to 59.6), natural products (46.2 to 81.7), and tourism & recreation indicators (42.6 to 59.9) scores from 2012 to 2019.

Based on the weighted average of the data listed in Table 1, the OHI index is calculated and reported as in Fig. 3.

Fig. 3 shows that in 2012, the OHI index score on marine life for the UAE was 75.62. There is a positive overall trend, and by 2019, the index score reached 81.80. There are only 18 countries that earned a score higher than the UAE. Most of the countries scored better than UAE are small island states with a sparsely located population. Notably, Germany has a rating of 86 and is ranked six^th^ globally. The global average index score is 71.

## 2 Experimental Design, Materials and Methods

The data is retrieved from 3 major sources, namely the UN sustainable development indicators, Bertelsmann-Stiftung SDG index, and Ocean Health Indicators. The UN and BS databases are structured to measure country-specific indicators for each goal. All databases are publicly available and can be downloaded directly from the respective websites. SDG 14 aims to sustain the quality of life on coastal ecosystems and open seas while providing sustainable income from fisheries.

As shown in Fig. 4, the data is filtered for only SDG 14, life below water. The filtering process is completed using the filter option under the Data tab in the Microsoft Excel package. The data in Excel is imported to the Tableau software (Tableau 2019.1) as an extracted data file. The visualizations are created within this software. The variable "year" is redefined as a date variable allowing Tableau to do time series analysis. All measures are automatically defined as numerical variables, and the measure names are defined as categorical variables. The parameters used in the design of the figures are as follows:

Columns: Year
Rows: Measure Values
Filters: Measure Names
Marks: Measure Names as Color and Measure Values as Text

Fig. 5 below shows a snapshot for the Tableau sheet design:

The Tableau outcome interface in Fig. 5 shows three worksheets that reflects the data from three sources. The visualizations can be used to see how marine sustainability indicators are changing over the last decade. The analysis is suitable for nations that apply policies to protect their coastal ecosystems [1]. It is also possible to use the same experimental design to make a comparative analysis of countries sharing similar ecosystems [15].

## Declaration of Competing Interest

The authors declare that they have no known competing financial interests or personal relationships which have, or could be perceived to have, influenced the work reported in this data article.

## Appendix Supplementary materials

Image, application 1
